# Stabilizing Perovskite Pb(Mg_0.33_Nb_0.67_)O_3_–PbTiO_3_ Thin Films by Fast Deposition and Tensile Mismatched Growth Template

**DOI:** 10.1021/acsami.3c16241

**Published:** 2024-02-29

**Authors:** Shu Ni, Evert Houwman, Nicolas Gauquelin, Dmitry Chezganov, Sandra Van Aert, Johan Verbeeck, Guus Rijnders, Gertjan Koster

**Affiliations:** † MESA+ Institute for Nanotechnology, Faculty of Science and Technology, University of Twente, Enschede 7500 AE, Netherlands; ‡ Electron Microscopy for Materials Research (EMAT), Department of Physics, University of Antwerp, Antwerpen BE-2020, Belgium; § NANOlab Center of Excellence, University of Antwerp, Antwerpen BE-2020, Belgium

**Keywords:** PMN−PT, kinetically stabilized perovskites, tensile mismatch template, relaxor ferroelectrics, Piezo-MEMS

## Abstract

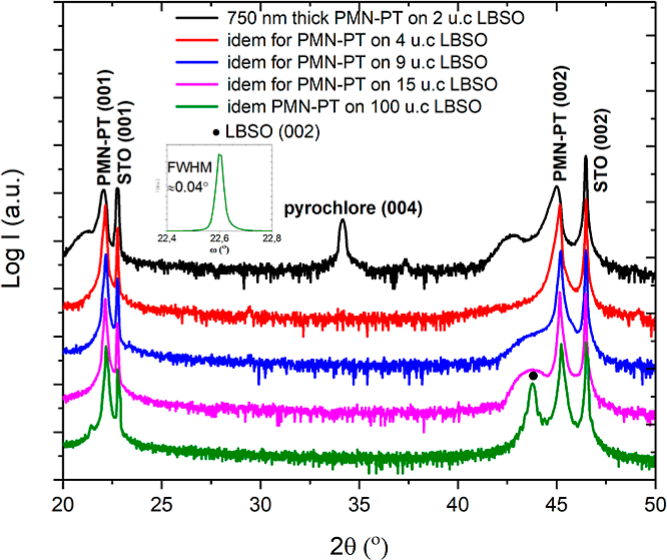

Because of its low hysteresis, high dielectric constant, and strong piezoelectric response, Pb(Mg_1/3_Nb_2/3_)O_3_–PbTiO_3_ (PMN–PT) thin films have attracted considerable attention for the application in PiezoMEMS, field-effect transistors, and energy harvesting and storage devices. However, it remains a great challenge to fabricate phase-pure, pyrochlore-free PMN–PT thin films. In this study, we demonstrate that a high deposition rate, combined with a tensile mismatched template layer can stabilize the perovskite phase of PMN–PT films and prevent the nucleation of passive pyrochlore phases. We observed that an accelerated deposition rate promoted mixing of the B-site cation and facilitated relaxation of the compressively strained PMN–PT on the SrTiO_3_ (STO) substrate in the initial growth layer, which apparently suppressed the initial formation of pyrochlore phases. By employing La-doped-BaSnO_3_ (LBSO) as the tensile mismatched buffer layer, 750 nm thick phase-pure perovskite PMN–PT films were synthesized. The resulting PMN–PT films exhibited excellent crystalline quality close to that of the STO substrate.

## 1 Introduction

Ferroelectric materials are widely utilized in different areas, such as PiezoMEMS, field-effect transistors (FETs), energy harvesting and storage, and haptic sensors and actuators.^1^ An important category of ferroelectric materials is formed by the relaxor ferroelectrics, which show a partially disordered structure and polar nanoregions (PNRs). A well-known type of such materials is lead magnesium niobate-lead titanate, mostly used in a composition close to the morphotropic phase boundary, [Pb(Mg_0.33_Nb_0.67_)O_3_]_0.67_–(PbTiO_3_)_0.33_ (PMN–PT 67/33), which in single crystal form exhibits a superior piezoelectric coupling coefficient and low hysteresis.^2^ Furthermore, PMN–PT exhibits a high dielectric constant, which makes it a promising gating layer for FETs, and it is believed that the integration of PMN–PT thin films on semiconductors like Si can potentially greatly enhance the performance of microelectronic devices.^3,4^ However, one of the major challenges of PMN–PT thin film synthesis is the concurrent growth of nonactive pyrochlore phases, such as the cubic Pb_3_Nb_4_O_13_, the rhombohedral Pb_2_Nb_2_O_7_, and the tetragonal Pb_3_Nb_2_O_8_, which degrades the performance of PMN–PT thin films significantly.^5^ Maria et al. reported the first attempt of epitaxial growth of [(Pb(Mg_0.33_Nb_0.67_)O_3_]_0.70_–(PbTiO_3_)_0.30_ (PMN–PT 70/30) on LaAlO_3_ substrates via pulsed laser deposition (PLD) using an oxygen/ozone gas mixture. It was found that avoiding the occurrence of pyrochlore phases is challenging, and perovskite PMN–PT (70/30) thin films can only be obtained within a narrow processing window. It also indicated that the epitaxial effect of LaAlO_3_ was insufficient to avoid the pyrochlore phases, and the electrical properties of the resulting films depended on the growth conditions.^6^ Bu et al. deposited phase-pure perovskite PMN–PT (67/33) thin films on vicinal SrTiO_3_ (STO) substrates, which had a large miscut angle of 8°.^7^ They proposed that the high density of steps on the vicinal substrate promoted the incorporation of volatile constituents, such as PbO, and thereby suppressed the formation of pyrochlore phases. Baek et al. integrated PMN–PT thin films on vicinal Si substrates using STO, deposited by reactive molecular beam epitaxy, as a template layer, while the PMN–PT was deposited by reactive sputtering.^4^ The resulting PMN–PT films demonstrated excellent piezoelectric coefficients and a high figure of merit for energy harvesting systems. Despite the excellent material and device performance, further investigation into the growth of PMN–PT on substrates with standard (low) miscut is needed for general device application. Boota et al. reported a systematic study on the processing conditions, such as the laser fluence, substrate temperature, target–substrate distance, and ambient gas pressure, for the deposition of PMN–PT (67/33) thin films on standard STO substrates (miscut∼ ± 0.1°).^5^ This study showed that the processing window is narrow and, secondly, that the observed self-bias field in the PMN–PT parallel plate capacitor devices depends on the fabrication conditions. The same authors integrated PMN–PT thin films on Si substrates using YSZ (yttrium-stabilized ZrO_2_) and CeO_2_ as the double layer buffer and SrRuO_3_ as the bottom electrode layer.^8^ The resulting PMN–PT thin films had a pure perovskite phase but showed varying strain states depending on the processing conditions. The same group showed that various Pb(Zr_1–*x*_,Ti_*x*_)O_3_ compositions can be used as a template layer for the growth of single-phase, epitaxial PMN–PT thin films, while at the same time introducing gigantic self-bias fields.^9,10^ Gabor et al. showed that a LaNiO_3_ (LNO) bottom electrode with a rough surface morphology can also help to stabilize the perovskite phase in PMN–PT thin films.^11^ They believe that the rough surface of LNO may enhance the sticking of Pb-based species during PMN–PT deposition. However, this also resulted in a PMN–PT thin film with a relatively large surface roughness. The same group also investigated the stoichiometric deviation between the ablated target and the resulting PMN–PT films and observed that the Pb and Mg contents exhibited the largest off-stoichiometry between the target and deposited layers. They concluded that stoichiometric transfer, albeit common, might not be guaranteed for the PLD process, especially for compositionally complicated material such as PMN–PT. The film with the highest piezoelectric response in this study was ablated from a ceramic target with 20% excess PbO.^12^ Kim et al. investigated the impact of epitaxial strain on the polar order of PMN–PT thin films by depositing 50 nm of phase-pure perovskite PMN–PT thin films on NdScO_3_(110), SmScO_3_(110), and GdScO_3_(110) substrates. The results demonstrated that compressive epitaxial strain reduced the polarization disorder, suppressing but not fully quenching the relaxor behavior of the PMN–PT thin films.^13^

Despite the extensive study and encouraging progress, the controlled fabrication of phase-pure perovskite PMN–PT layers remains challenging, and there is limited insight into the underlying mechanism of the successful growth. Many reported PMN–PT growth studies using PLD rely on targets with additional Pb and substrates with high-miscut or rough surface, as the volatility and re-evaporation of the Pb during the deposition are believed, by many authors, to be the cause for the nucleation of the passive pyrochlore phases.^4,5,7−11,13^ However, there is a significant variation in the target stoichiometry and growth conditions in the literature, and the mechanism of modified growth parameters on the stability of perovskite phase of PMN–PT remains largely elusive. Here, we report on the impact of deposition rates and mismatch strain on the synthesis of PMN–PT thin films on STO substrates using PLD. We observe that a higher deposition rate enhances the mixing of B-site cations, such as Mg, in the initial layer of PMN–PT (∼15 nm) and accelerates the relaxation of the compressive strain induced by the STO substrate to occur within the first layers of the deposit. As a result, we speculate that these effects help to suppress the nucleation probability of the passive phases. Furthermore, the PMN–PT layers with secondary passive phases still contain a significant Pb fraction, implying that the re-evaporation of Pb species might not be the primary factor for the phase segregation. By combining the use of La-doped BaSnO_3_ (LBSO ) as the template layer with rapid laser ablation, the epitaxial phase-pure perovskite PMN–PT films of 750 nm were deposited, and the resulting films exhibit excellent crystalline quality. The tensile mismatched template layer and the high deposition rate are shown to contribute to improved mixing of B-site cations such as Mg in the initial PMN–PT layer.

## 2 Experimental Methods

### 2.1 Sample Preparation

To gain insights into the possible formation mechanism of pyrochlore phases, PMN–PT thin films were first grown directly on TiO_2_- terminated STO (100) substrates. The substrate treatment procedure is described in ref (14). The PMN–PT thin films were deposited by PLD using a KrF Excimer laser operating at 248 nm. A commercial phase-pure perovskite [Pb(Mg_0.33_Nb_0.67_)O_3_]_0.67_–(PbTiO_3_)_0.33_ (PMN–PT 67/33) ceramic target without an additional Pb component was used for the laser ablation. A laser fluence of 2.0 J cm^–2^ and ablation frequencies of 1, 5, 10, and 20 Hz were used. The growth temperature was at 600 °C and the oxygen pressure was at 0.25 mbar. The spot size was 1.77 mm^2^ and the target–substrate distance was 50 mm. Based on the results of PMN–PT growth on STO, LBSO thin films were grown on the STO substrate to stabilize the perovskite phase of PMN–PT, which simultaneously function as the bottom electrode layer in capacitor structures. A commercial ceramic La_0.07_Ba_0.93_SnO_3_ target was used and the LBSO growth was optimized with respect to surface morphology, crystalline quality, and conductivity, using laser fluence (1.3 J cm^–2^), repetition rate (1 Hz), and spot size (0.59 mm^2^) as the optimization parameters. The growth temperature and oxygen pressure were fixed at 830 °C and 0.13 mbar, respectively. PMN–PT thin films of 750 nm were subsequently grown on the LBSO template (electrode) layer using the same deposition conditions as described above. To characterize the electrical responses of the PMN–PT films, a 50 nm thick SrRuO_3_ (SRO) layer was deposited as the top electrode for some samples. The SRO layer was grown from a ceramic SrRuO_3_ target using a laser fluence of 2.0 J/cm^2^, a deposition rate of 4 Hz, and an oxygen background pressure of 0.25 mbar at a growth temperature of 600 °C. The layer stacks were cooled at 100 mbar of oxygen after deposition. The top electrode was patterned by photolithography and Ar- ion beam etching to form parallel plate capacitor structures with a 200 × 200 μm^2^ area.

### 2.2 Sample Characterization

During the deposition of the LBSO, PMN–PT, and top SRO layers, the surface morphology and crystal structure of the deposited layers were monitored in situ via reflection high-energy electron diffraction (RHEED). After deposition, the crystallographic properties of the thin films were characterized by X-ray diffraction (XRD, Panalytical MRD), and the surface morphology was characterized by atomic force microscopy (AFM. Bruker). The chemical and structural properties of the initial growth layers of PMN–PT were visualized by Cs-corrected scanning transmission electron microscopy (STEM) equipped with a super-X EDX detector. The microscope was operated at 300 kV with a 20 mrad convergence angle. The electrical responses of the PMN–PT heterostructure were characterized with an aixACCT TF-2000 analyzer, combined with a double beam laser interferometer for displacement measurements. In the electrical measurements, a triangular bipolar excitation voltage wave, scanning at a frequency of 1 kHz, was used. In all electrical measurements, the bottom electrode was grounded using silver paste that was applied at the edge of the substrate. The top electrode and the silver paste were contacted by metal probes.

## 3 Results and Discussion

### 3.1 Growth of PMN–PT Thin Films on STO Substrates

**Figure 1:**
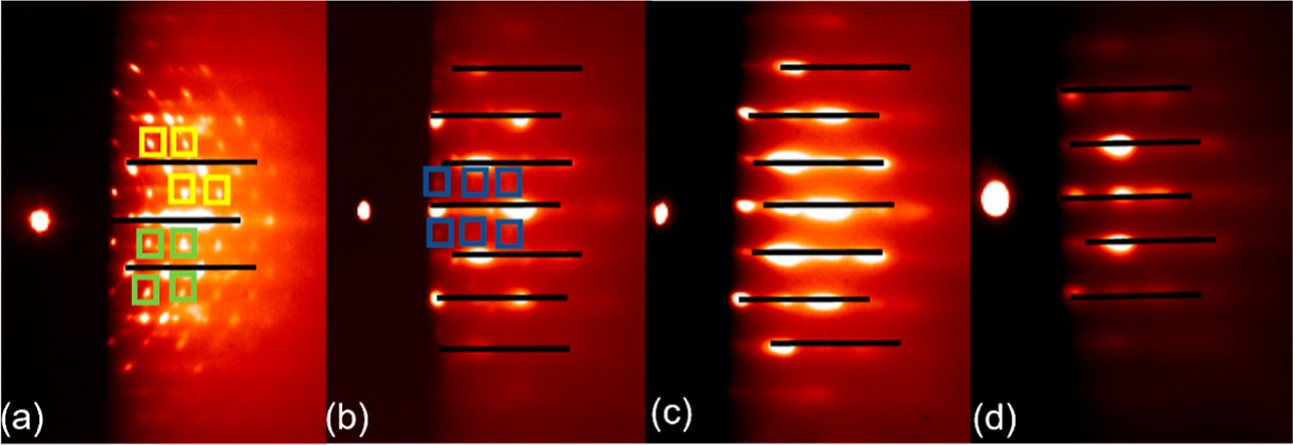
RHEED profiles of 15 nm PMN–PT films deposited on the STO substrates using ablation frequencies of 1 (a) 5 (b) 10 (c), and 20 Hz (d). Squares correspond to the RHEED patterns of secondary (pyrochlore) phases and the lines correspond to the perovskite phase.

In the first series of experiments, 15 nm thick PMN–PT thin films were directly deposited on STO substrates using laser ablation frequencies of 1, 5, 10, and 20 Hz, respectively. The RHEED patterns of the resulting films are given in Figure 1. From the RHEED pattern of the film deposited at 1 Hz (Figure 1a), it is concluded that this film contains material with different crystal symmetries (as indicated by the squares), which are ascribed to nonperovskite phases in the PMN–PT film. The pattern of the film deposited at 5 Hz (Figure 1b) shows that the film is predominantly made up of the perovskite phase (indicated by the black lines), but a secondary phase is also visible (indicated by the squares). The RHEED patterns of the films deposited at 10 and 20 Hz (Figure 1c,d) indicate a pure perovskite phase.

**Figure 2:**
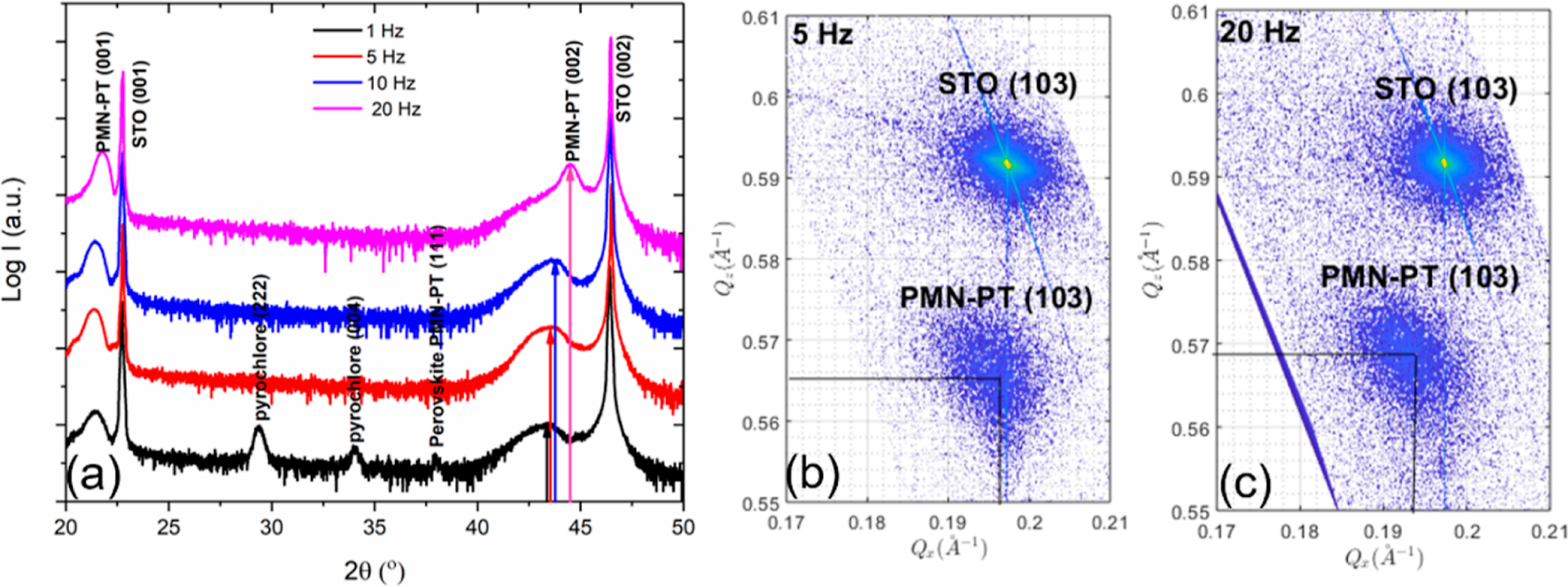
XRD scans of PMN–PT grown with different repetition rates. (a) XRD θ–2θ scans of 15 nm thick PMN–PT films grown on the STO substrates deposited with different laser pulse repetition rates. (b) Reciprocal space map around (103) peaks of PMN–PT grown on STO at 5 Hz. (c) Idem for the PMN–PT grown at 20 Hz.

To gain more insights into the crystallographic properties, XRD was performed. The XRD θ–2θ scan in Figure 2a shows that the film grown at 1 Hz contains pyrochlore phases, whereas other films appear to be of a pure perovskite phase. Probably because of the small volume fraction, the secondary phase appearing in the RHEED pattern does not show up in the XRD scan for the PMN–PT film deposited at 5 Hz. The position of the (002) reflection of the perovskite PMN–PT shifts to a slightly larger angle with increasing deposition frequency. To further investigate this effect, reciprocal space mapping (RSM) was performed on the samples that were fabricated at 5 and 20 Hz (Figure 2b,c). The deduced (tetragonal) room-temperature lattice parameters and the pseudocubic lattice parameter of the PMN–PT are found to be, respectively, *a* = 3.920 Å, *c* = 4.083 Å, and *a*_pc_ = 3.974 Å for the film deposited at 5 Hz, and *a* = 3.995 Å, *c* = 4.059 Å, and *a*_pc_ = 4.016 Å for the 20 Hz film. We conjecture that the differences in the lattice parameters are associated with substrate-induced compressive in-plane strain in the PMN–PT, as we will corroborate below. The STO substrate has a room temperature lattice parameter of 3.905 Å and a lattice parameter of 3.930 Å at the deposition temperature of PMN–PT (see Figure S1). This gives rise to a compressive epitaxial lattice mismatch of −2.5% and therefore a compressive in-plane strain in the initial layer of the PMN–PT (with a pseudocubic lattice parameter of 4.029 Å at deposition temperature using the thermal expansion coefficient of PMN–PT of 3.2 × 10^–6^ °C^15−17^). This large compressive in-plane strain induced in the PMN–PT by the STO substrate is expected to reduce the random distribution of the B-site atoms in the PMN–PT layer, which in turn is expected to lead to phase segregation and thereby attributes to the nucleation of the pyrochlore phases.^2,4,18^Figure 2a shows that the films grown at 10 Hz or less all show similar PMN–PT reflections: a broad peak with an average room temperature in-plane lattice parameter close to that of the substrate (*a* = 3.920 Å for the RSM of the 5 Hz film shown in Figure 2b), while the out-of-plane lattice parameter (*c* = 4.083 Å) is larger than the pseudocubic, room-temperature lattice parameter of bulk single crystal PMN–PT (*a*_pc_ = 4.022 Å). The pseudocubic lattice parameter of the strained layer is also strongly reduced to 3.974 Å, indicating a significant decrease in the unit cell volume for the 5 Hz film. For the film grown at 20 Hz, a sharp peak on top of the broad strained film peak is observed. From the RSM, more relaxed lattice parameters (*a* = 3.995 Å and *c* = 4.059 Å) and a pseudocubic lattice parameter (*a*_pc_ = 4.016 Å) were deduced. The *a*_pc_ is close to the bulk value, indicating that under these conditions, the film is nearly fully relaxed. Furthermore, we see for the film grown at 1 Hz significant pyrochlore phase peaks, which are not visible in the films grown at higher rates. These results indicate that the strain state and the crystalline quality of the deposited PMN–PT films (15 nm) can be tuned by applying different deposition rates. A high deposition rate results in a PMN–PT film which is nearly relaxed and also suppresses the nucleation and growth of pyrochlore phases, enhancing the crystalline quality of the perovskite PMN–PT phase.

**Figure 3:**
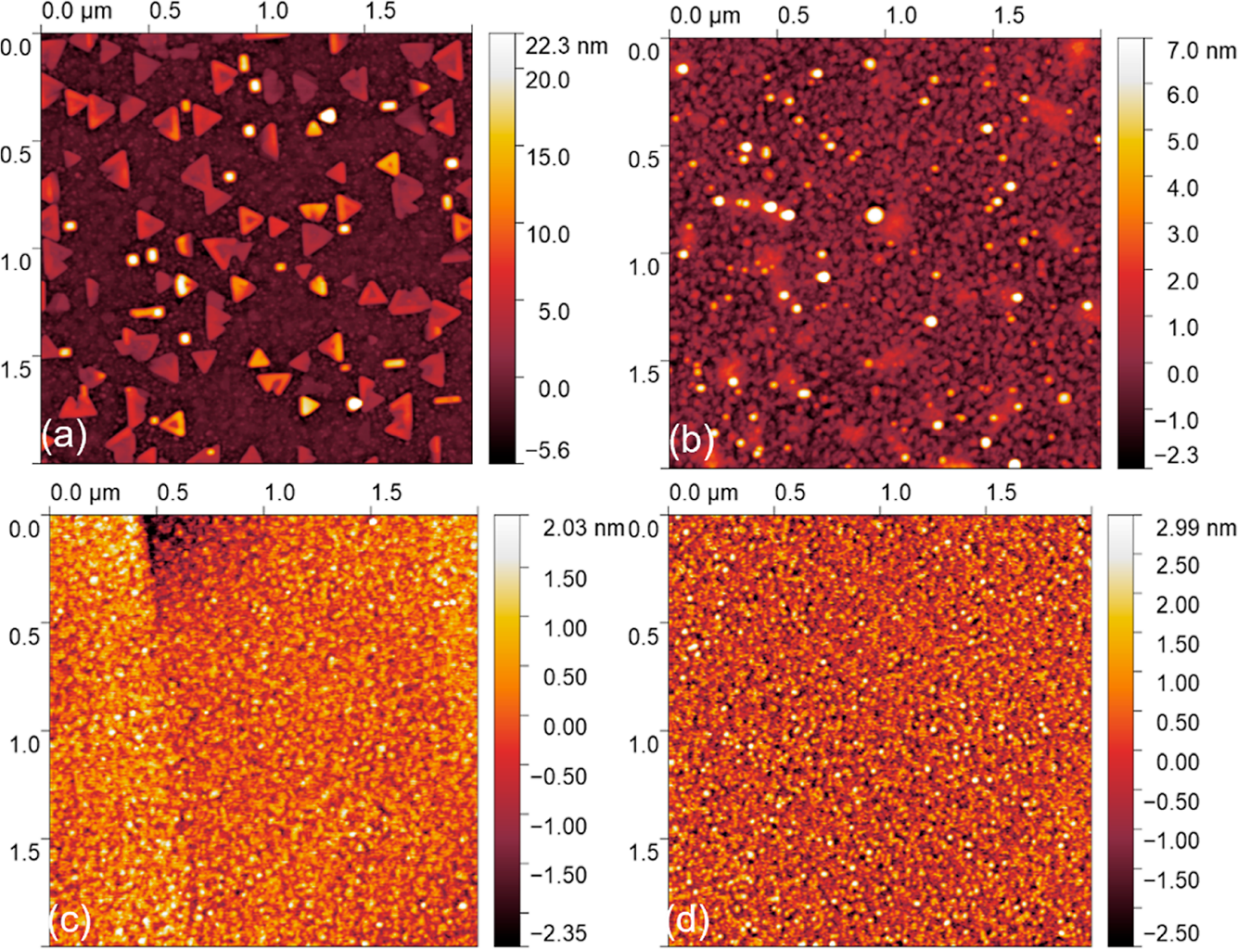
AFM images of 15 nm of PMN–PT films deposited on the STO substrates using ablation frequencies of (a) 1, (b) 5, (c) 10, and (d) 20 Hz.

The AFM images of the surfaces of these films are shown in Figure 3. The surface of the 1 Hz film shows clusters of material with triangular and rectangular morphology, attributed to the formation of the nonperovskite phases. For the film grown at 5 Hz, particulates are observed on the surface and are believed to be (part of) the nuclei of the pyrochlore phases. The films deposited by using 10 and 20 Hz exhibit clean and smooth surfaces with a root mean square roughness of 0.6 and 1.0 nm, respectively.

**Figure 4:**
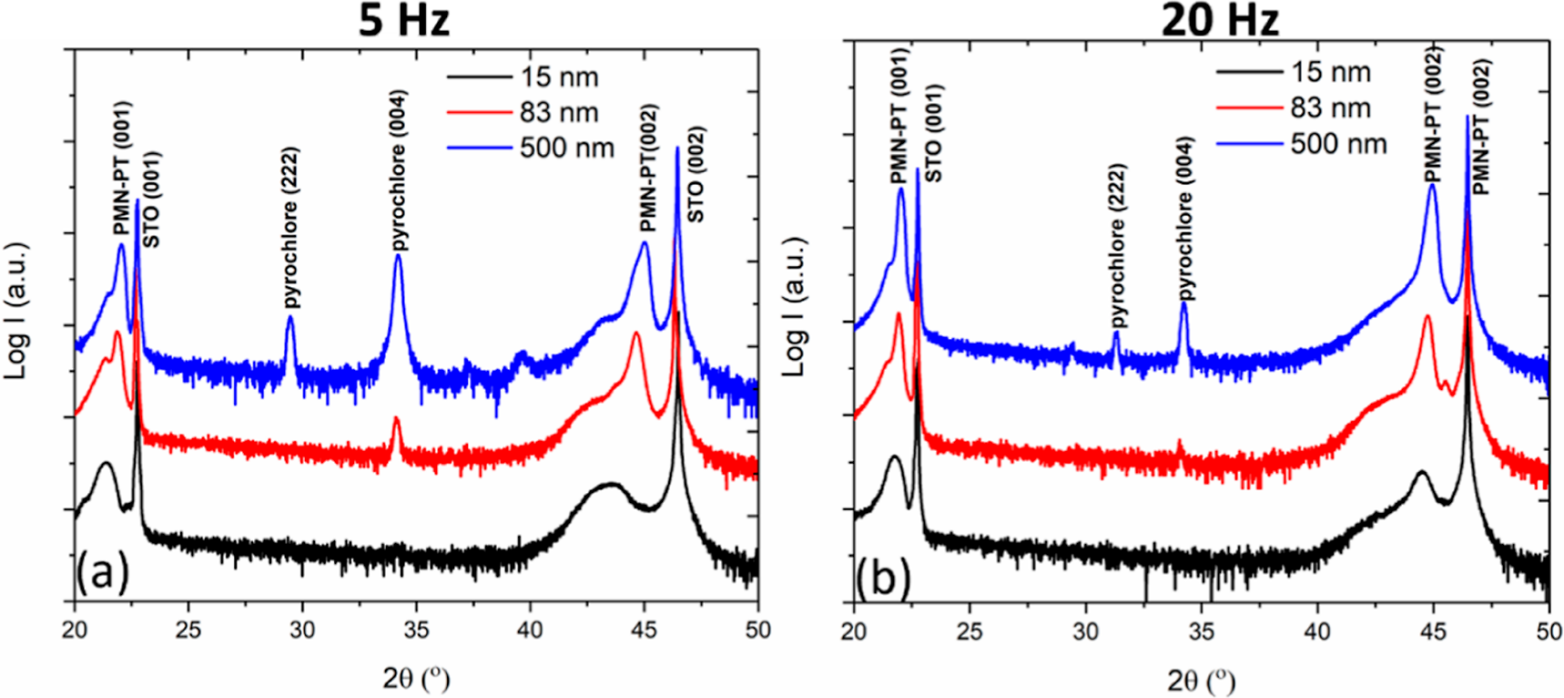
XRD scans of PMN–PT films grown on STO using different deposition frequencies (a) θ–2θ scans of 15, 83, and 500 nm thick PMN–PT films on STO substrates deposited at 5 Hz. (b) Idem for 15, 83, and 500 nm films deposited at 20 Hz.

**Figure 5:**
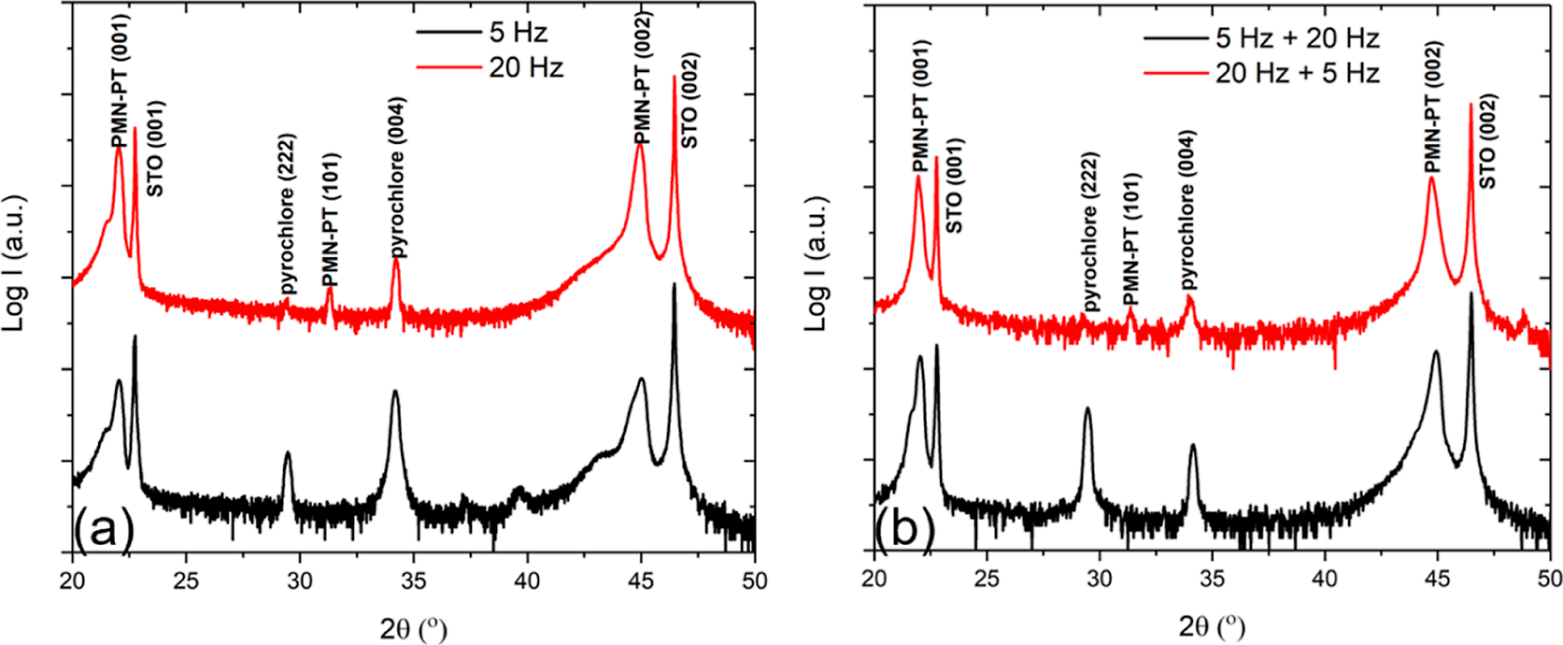
XRD scans of PMN–PT thin films grown on STO, of which the initial layer was deposited with different frequencies: (a) θ–2θ scans of 500 nm thick PMN–PT films grown at 5 and 20 Hz; (b) θ–2θ scans of 500 nm thick PMN–PT films, of which the initial growth layer was grown at 5 and 20 Hz, followed by the growth of 485 nm thick PMN–PT using 20 and 5 Hz, respectively.

In a second series of experiments, we investigated the thickness dependence of the crystalline properties of PMN–PT films grown at different deposition rates. Figure 4a shows the θ–2θ scans of 15, 83, and 500 nm thick films grown at 5 Hz. It is seen that a pyrochlore phase appeared in the 83 nm thick film, and its volume fraction increased in the 500 nm thick film. Figure 4b shows the scans for films grown at 20 Hz. In this, the pyrochlore phases only appear in the thickest film (500 nm), and only a hint of a pyrochlore phase is present in the 83 nm thick film. Compared with the films of the same thicknesses grown at 5 Hz, the volume fraction of the pyrochlore phases is much smaller for the films grown at 20 Hz, again showing that a high deposition rate suppresses the growth of the pyrochlore phases. We speculate that a higher deposition rate reduces the effective surface diffusion and nucleus ripening during deposition and, thereby, enhances the local mixing and disorder of the PMN–PT film. This suppresses the segregation of B-site elements, which is known to be the cause of the presence of pyrochlore phases in PMN–PT bulk ceramics.^2,4^ However, even with a high deposition rate of 20 Hz, it is found that pyrochlore phases are part of the films grown on STO. To gain more insights into the influence of the initial growth layer, 15 nm PMN–PT films were first grown on STO substrates using deposition rates of 5 and 20 Hz, followed by the growth of 485 nm PMN–PT layers using 20 and 5 Hz, respectively. The XRD scans (Figure 5b) of these two films, PMN–PT(5 + 20) and PMN–PT(20 + 5), show that the volume fraction of the pyrochlore phases is much larger for the PMN–PT(5 + 20) film. This indicates that the relative volume fraction of the pyrochlore phases in the PMN–PT thin film is determined by the initial growth layer. Furthermore, it shows that it is not possible to suppress the further growth of the pyrochlore phases via accelerated deposition after they have already nucleated in the initial growth layer.

**Figure 6:**
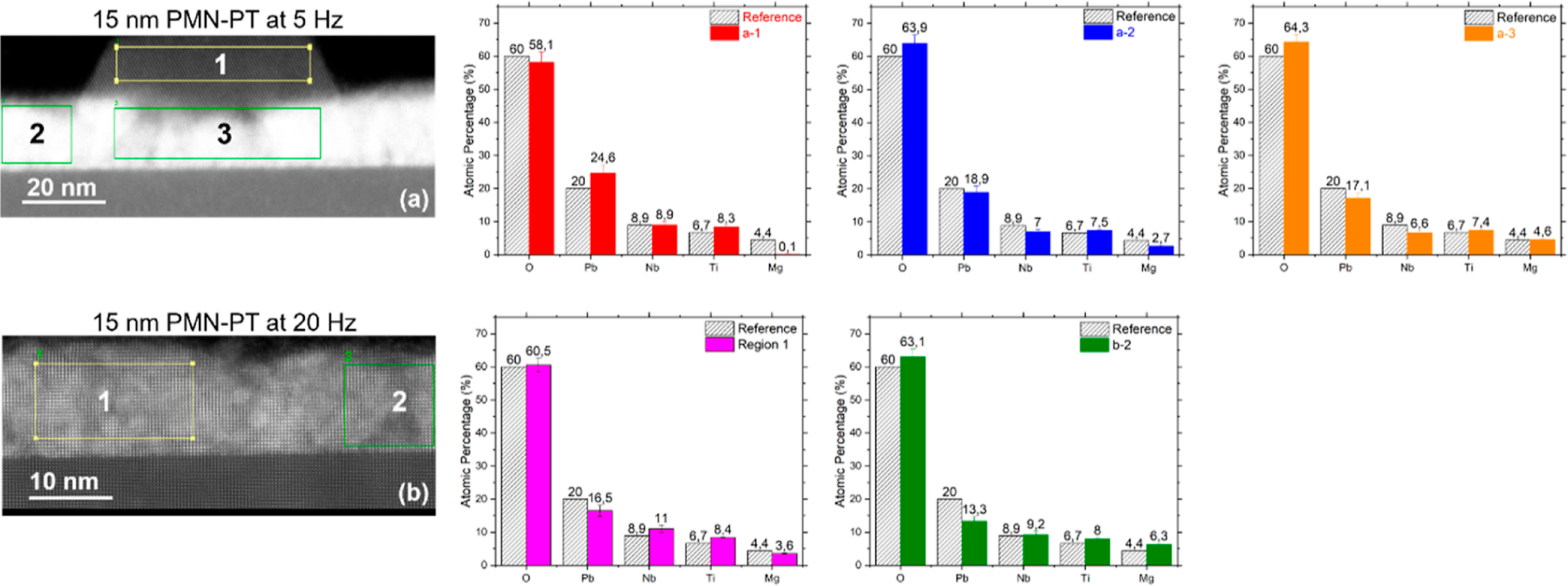
STEM micrographs and atomic fraction of elements of 15 nm thick PMN–PT layers grown at 5 and 20 Hz. (a,b) Angular dark-field image (a-1,-2,-3) atomic fraction of the constituent elements at regions 1, 2, and 3 of PMN–PT film deposited at 5 Hz (b-1,-2) atomic fraction of constituent elements at regions 1, 2, and 3 of PMN–PT film deposited at 20 Hz. Dashed columns refer to the atomic percentage in the stoichiometric composition of [Pb(Mg_0.33_Nb_0.67_)O_3_]_0.67_–(PbTiO_3_)_0.33_.

STEM was employed to further investigate the structural and chemical properties of the initial PMN–PT layers deposited at 5 and 20 Hz. From Figure 6a, it is seen that the film deposited at 5 Hz contains pyramid-shaped structures, which are attributed to the pyrochlore phases. This is consistent with our RHEED and AFM data shown in Figures 1 and 3. Secondary phases are not observed for the film deposited at 20 Hz (Figure 6b). However, extended defects are seen in this film, which are believed to be caused by the large lattice mismatch between PMN–PT and the STO substrate. Energy-dispersive X-ray spectroscopy (EDX) was employed to characterize the chemical composition variations across the different regions of the film. Figures S2–S6 show the chemical maps, and Figure 6a-1–a-3 gives the normalized atomic fraction of the composing elements in different sections of the PMN–PT film deposited at 5 Hz. From Figure 6a-1, we can see that the pyrochlore region (region 1) has only traces of Mg but a significantly higher fraction of Pb than the nonpyrochlore regions (regions 2 and 3) for the film deposited at 5 Hz. The high Pb content of the pyrochlore region suggests that the volatility, i.e., pronounced re-evaporation of Pb during the deposition, might not be the major cause for the formation of pyrochlore phases. For the film deposited at 20 Hz, the region that contains extended defects (Figure 6b) was found to be slightly richer in Mg (∼6.3 at. %, Figure 6b-2) in combination with a lower Pb component than the film deposited at 5 Hz. From the STEM study, we propose that the formation of pyrochlore phases is associated with clustering of B-site cations, such as Mg, instead of the commonly believed loss of Pb (A-site cation) during the PMN–PT growth.^4,5,7,11^ This clustering might create localized strain, structural distortion, and defects and eventually collapse the perovskite phase and the formation of pyrochlore phases.

### 3.2 Growth of the PMN–PT Thin Films on STO Substrates Using LBSO as a Buffer Bottom Electrode

**Figure 7:**
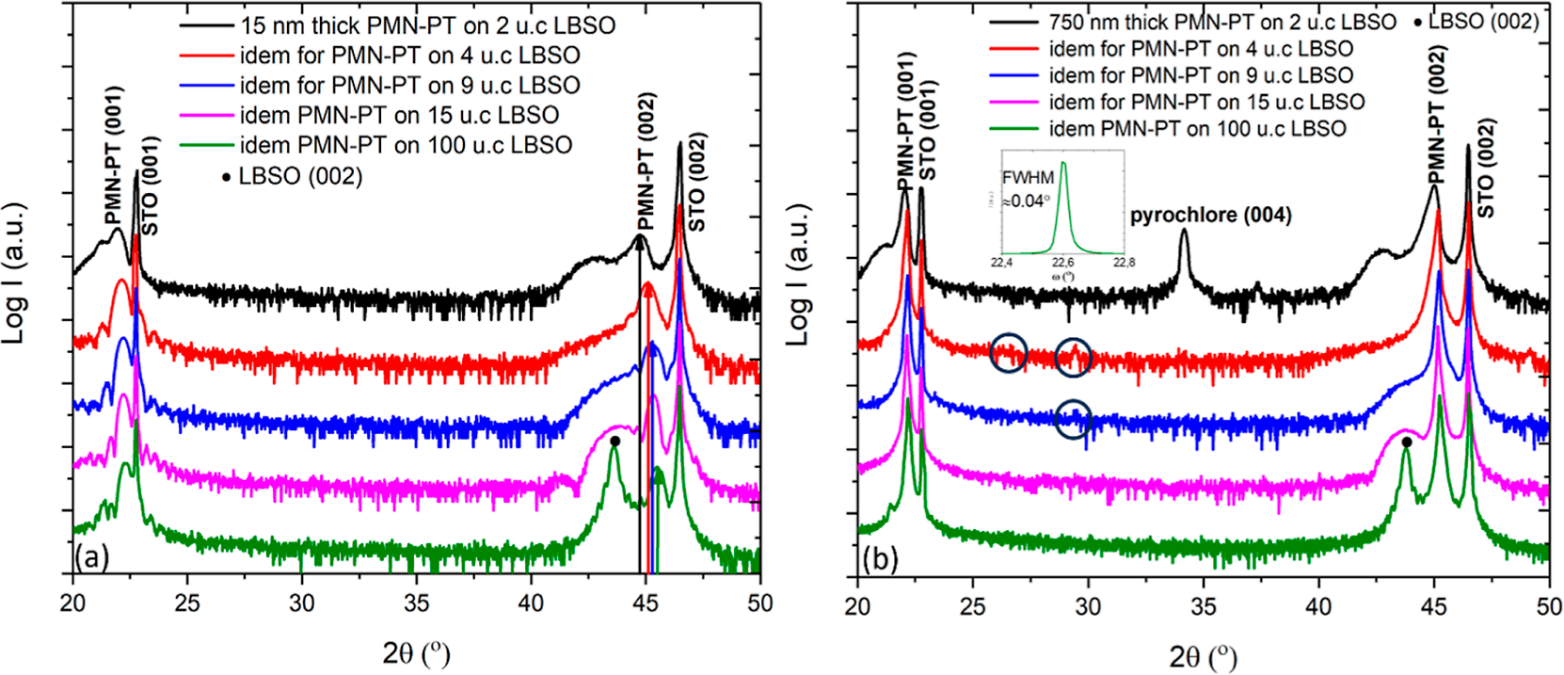
XRD scan of 15 and 750 nm thick PMN–PT films grown on LBSO of 2, 4, 9, 15, and 100 u.c. (a) θ–2θ scans of 15 nm thick PMN–PT thin films grown on LBSO of different thicknesses. (b) Idem for 750 nm thick PMN–PT thin films. The inset is ω scan of (002) reflection of 750 nm PMN–PT film on 100 u.c. LBSO. Dashed columns refer to the atomic percentage of stoichiometric [Pb(Mg_0.33_Nb_0.67_)O_3_]_0.67_–(PbTiO_3_)_0.33_.

Often, SrRuO_3_ (SRO) is used as the bottom electrode in ferroelectric capacitor devices. SRO strains nearly fully to the underlying film or substrate; hence, one can expect that PMN–PT films grown on SRO/STO substrates show the same features as the films discussed above. This is indeed observed in our study (Figure S7). Based on the assumption that the formation of pyrochlore phases arises from a large compressive lattice mismatch in the initial growth layer of the PMN–PT, a bottom electrode material with a room-temperature lattice parameter [*a*_pc_(RT) = 4.11 Å] that is larger than that of the PMN–PT was chosen to introduce a template layer that shows a tensile lattice mismatch (+2.2%). LBSO films of 2, 4, 9, 15, and 100 unit cell (u.c.) were deposited on STO substrates. The surface morphologies of the grown LBSO thin films are shown in Figure S8. Subsequently, the PMN–PT films of 15 and 750 nm were deposited using an ablation frequency of 20 Hz. The RHEED profiles of the 15 nm thick PMN–PT films on the LBSO films of different thicknesses are shown in Figure S10, indicating that all films consist of pure perovskite phase. The XRD θ–2θ scans of these films (Figure 7a) are the same. For the PMN–PT grown on 2 u.c. thick LBSO, two (002) peaks of perovskite PMN–PT are observed, which are attributed, respectively, to the more compressively strained initial growth layer and the partially relaxed PMN–PT layer. The LBSO layer is too thin to be observed, and the peak profile of PMN–PT from this sample is similar to that of the 15 nm thick PMN–PT film directly grown on STO at 20 Hz (Figure 4b), indicating that the deposited PMN–PT films have similar strain states. With increasing thickness of the LBSO layer, the position of the (002) reflection of the perovskite PMN–PT shifts continuously to a higher angle, and the diffraction peak becomes sharper, implying that the strain state of the initial growth layer of the PMN–PT changes continuously to a more relaxed state (Figure 7a). Furthermore, the LBSO reflection becomes stronger and is clearly seen for 15 and 100 u.c. LBSO layers. It is known that LBSO tends to relax with increasing thickness by forming thread dislocations.^18,19^ As a result, PMN–PT films grown on thicker LBSO are expected to be under less compressive strain, which reduces the atomic segregation and thereby enhances its crystalline quality.

**Figure 8:**
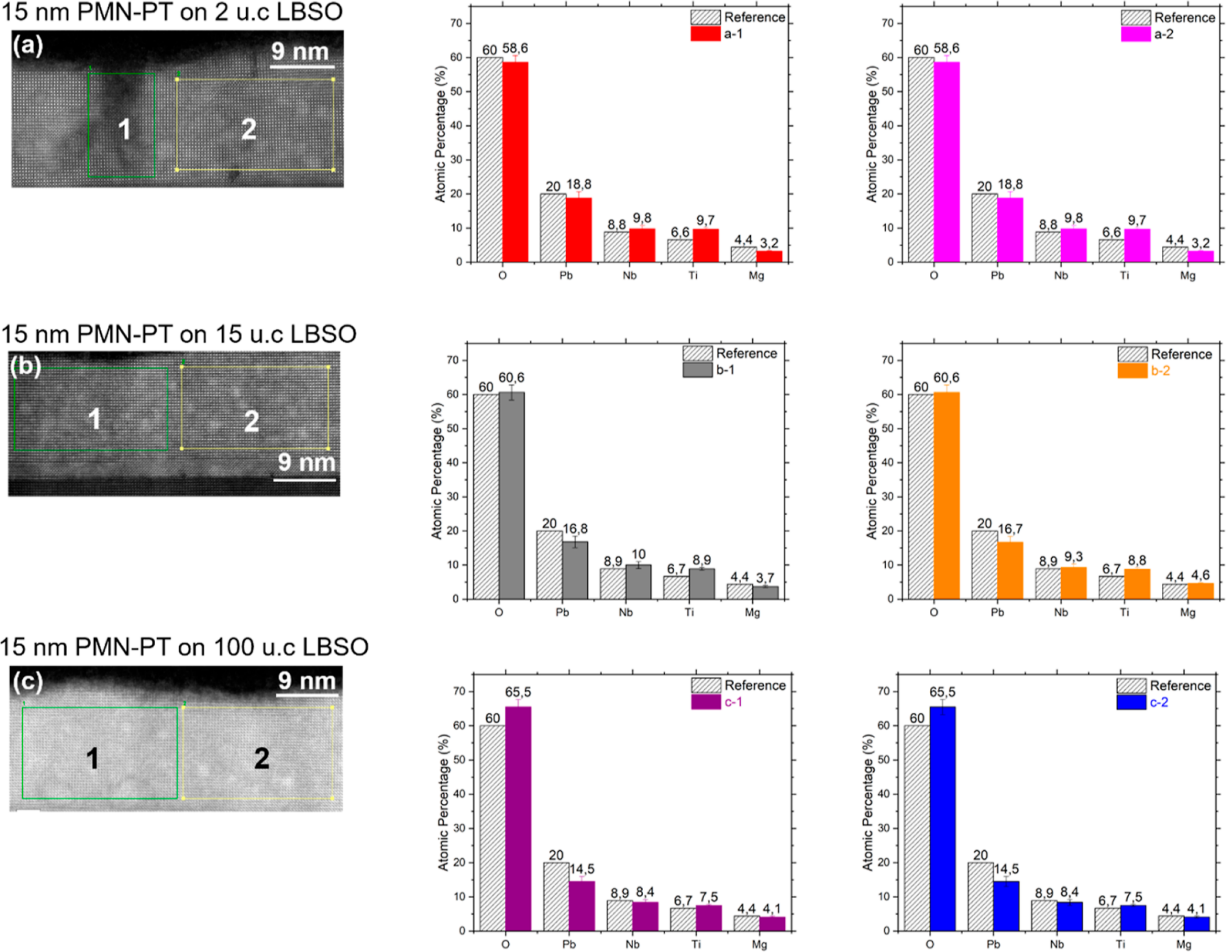
STEM micrographs and atomic element fraction of 15 nm thick PMN–PT layers grown on 2, 15, and 100 u.c. thick LBSO buffer electrode at 20 Hz. (a–c) Angular dark-field image of 15 nm thick PMN–PT on 2, 15, and 100 u.c. LBSO (a-1,-2), (b-1,-2), and (c-1,-2) atomic fraction of constituent elements at different regions. Dashed columns are the atomic percentage in the stoichiometric composition of [Pb(Mg_0.33_Nb_0.67_)O_3_]_0.67_–(PbTiO_3_)_0.33_.

STEM measurements were performed on the 15 nm thick PMN–PT layers grown on LBSO of 2, 15, and 100 u.c. As shown in Figure 8, there are no secondary phases in these samples. Nonetheless, extensive defects are observed in the PMN–PT film grown on the 2 u.c. thick LBSO film, which are believed to be strain-induced dislocations as the 2 u.c. thick LBSO is nearly fully strained to the STO substrates (Figures 8a and S2). The density of these defects decreases for the PMN–PT layer grown on a thicker LBSO, as shown in Figure 8b (PMN–PT layer on 15 u.c. LBSO) and Figure 8c (PMN–PT layer on 100 u.c. LBSO).

EDX was employed to obtain the chemical composition of the deposited film, and the results are given in Figure 8a-1,a-2,b-1,b-2,c-1,c-2. It is seen that the defective region (region 1 in Figure 8a) contains slightly more Mg. The thin PMN–PT layers deposited on 15 and 100 u.c. LBSO show improved homogeneity of Mg.

**Figure 9:**
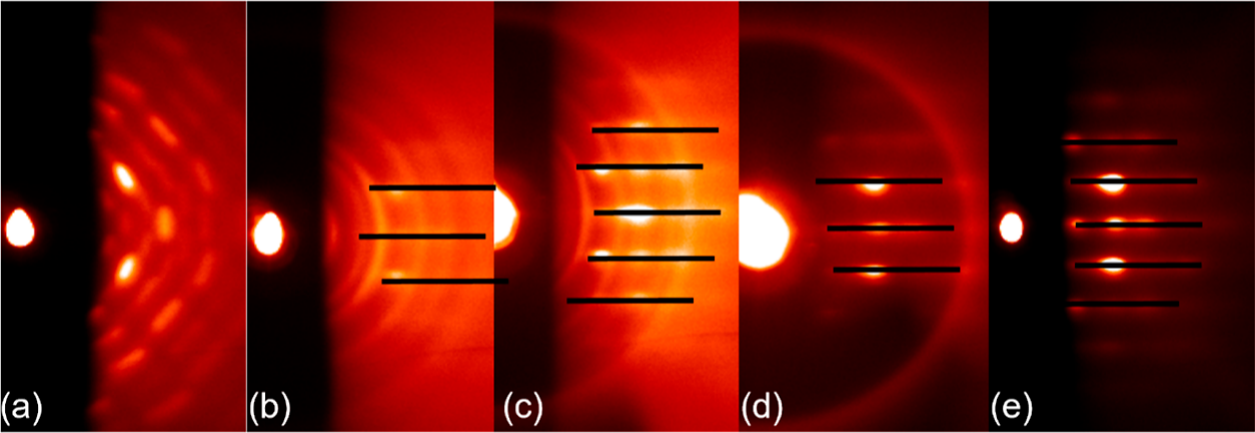
RHEED profiles of 750 nm of PMN–PT films deposited on LBSO of (a) 2, (b) 4, (c) 9, (d) 15, and (e) 100 u.c. Lines correspond to the RHEED pattern of perovskite phase of PMN–PT.

Also, 750 nm PMN–PT thick films were deposited on the LBSO electrode layers with different thicknesses. The XRD θ–2θ scans of the resulting films are given in Figure 7b. The PMN–PT film grown on 2 u.c. LBSO contains a pyrochlore phase, and a hint of the pyrochlore phases (highlighted by the blue circles) is observed in the XRD scans of the PMN–PT films grown on 4 and 9 u.c. thick LBSO. PMN–PT layers deposited on 15 and 100 u.c. LBSO consist of the phase-pure perovskite phase. Also, the RHEED patterns of these films (Figure 9a–c) show that nonperovskite phases are formed in PMN–PT films on 2, 4, and 9 u.c. LBSO, and the films grown on 15 and 100 u.c. LBSO appear to consist of the pure perovskite phase. It is also observed that the intensity of the perovskite PMN–PT pattern (indicated by the black lines) increases with thicker LBSO.

**Figure 10:**
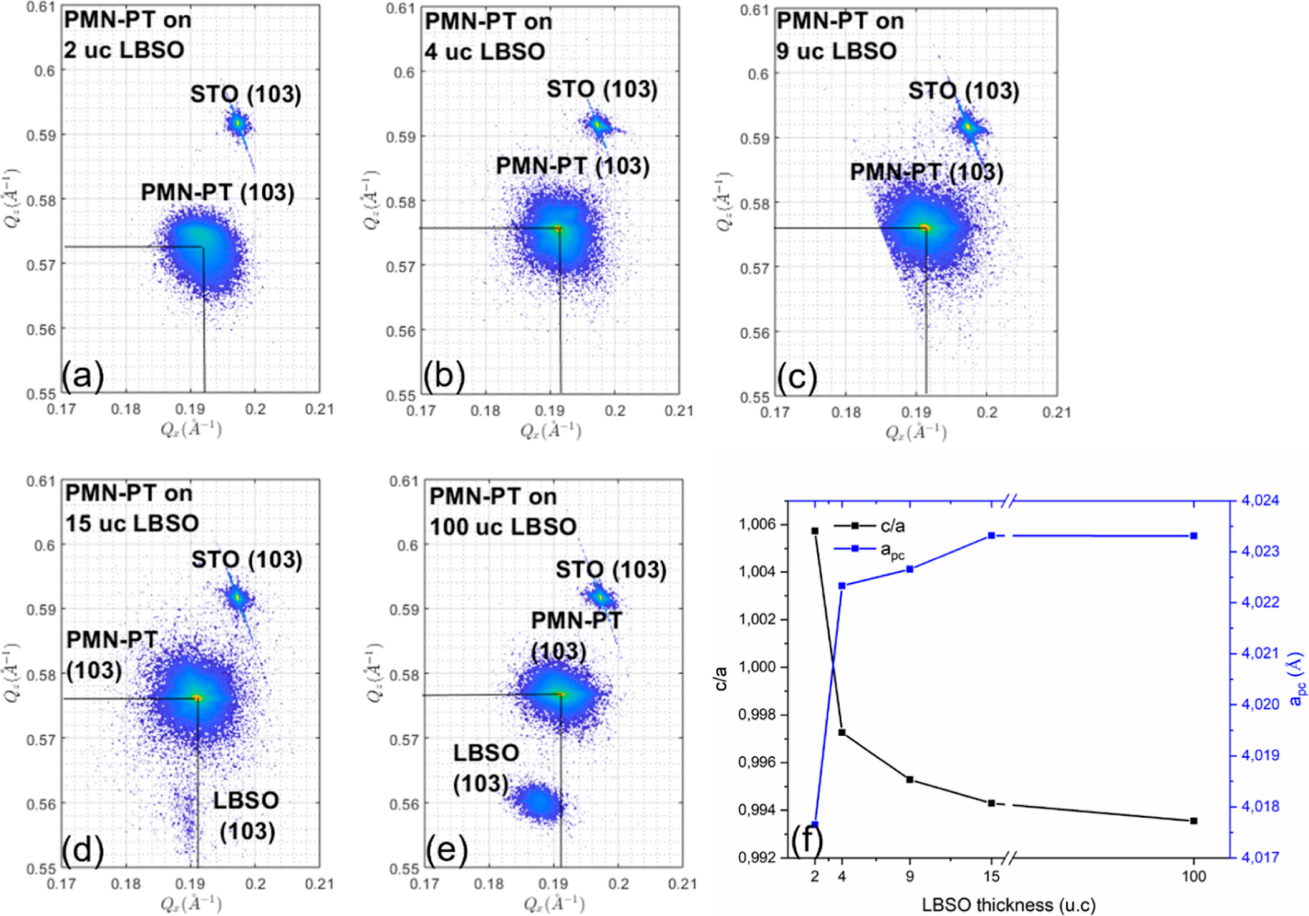
Reciprocal space maps around (103) peaks of 750 nm thick PMN–PT films grown on LBSO of (a) 2, (b) 4, (c) 9, (d) 15, (e) 100 u.c., and (f) deduced tetragonality and pseudocubic lattice parameters of 750 nm thick PMN–PT grown on LBSO of different thicknesses.

The inset of the Figure 7b shows the ω scan of the (002) reflection of PMN–PT on 100 u.c LBSO. The fwhm of the rocking curve is about 0.04°, which is an order of magnitude less than the single crystal value and close to the value of our STO substrate and the best value for a thin film reported in the literature.^12^ We note that the fwhm’s of the substrates used in literature^12^ are an order of magnitude less than those of our STO substrates. The RSMs of the 750 nm PMN–PT films on LBSO with different thicknesses are shown in Figure 10, from which the tetragonality (a,c) and pseudocubic lattice parameters are deduced. The tetragonality of the PMN–PT films decreases with increasing LBSO thickness (Figure 10f), indicating that the resulting PMN–PT films are under increasing tensile strain. Additionally, the pseudocubic lattice parameters (and thus the unit cell volume) of the PMN–PT films also increase with increasing LBSO thickness, approaching the pseudocubic bulk value for LBSO thicknesses larger than 4 u.c.’s.

### 3.3 Ferroelectric and Piezoelectric Properties of the Phase-Pure Perovskite PMN–PT Films on LBSO

**Figure 11:**
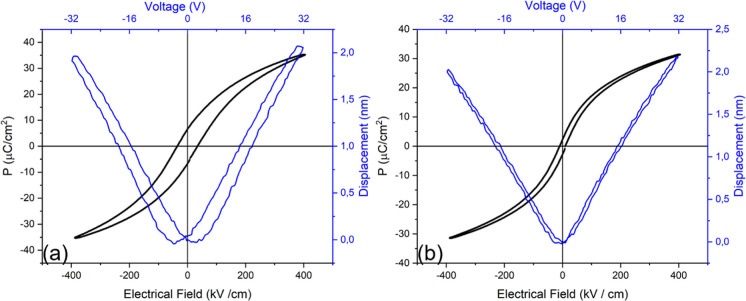
Polarization and displacement responses of (a) SRO/PMN–PT/LBSO heterostructure and (b) PMN–PT heterostructure with engineered electronic contact.

Figure 11a shows the polarization-electric field (P-E) and displacement-electric field (D-E) responses of a SRO/PMN–PT/LBSO capacitor. The thickness of SRO, PMN–PT, and LBSO is 50, 750, and 41 nm (100 u.c), respectively. The P-E loop is slanted and symmetrical and shows significant hysteresis with a coercive field of about 35 kV/cm. The remanent polarization is about 5 μC/cm^2^ and the piezoelectric coefficient is approximately 70 pm/V. The hysteresis of the P-E and D-E responses is thought to arise from the exchange of electronic charges between the LBSO and the initial growth layer of the PMN–PT. Nearly, hysteresis-free P-E and D-E responses can be achieved by introducing an additional SRO layer of 2 nm between the LBSO bottom electrode and the PMN–PT layer. Figure 11b shows the P-E and D-E responses of an SRO/PMN–PT/SRO/LBSO capacitor, where the layer thicknesses are 50, 750, 2, and 41 nm, respectively. Detailed information on the electronic contact engineering is described in our earlier work.^20^

## 4 Conclusions

Epitaxial growth of 750 nm thick, phase-pure, perovskite PMN–PT thin films on STO substrates was achieved by PLD with a high deposition rate (20 Hz) and by making use of a template layer (LBSO). Direct growth of PMN–PT on STO substrates resulted in films that contain a significant volume fraction of passive pyrochlore phases. It is found that an increased deposition rate promotes mixing of the B-site cations and relaxation of the compressive mismatch strain in the PMN–PT initial growth layer, which suppresses but does not fully prevent the nucleation of pyrochlore phases. PMN–PT thin films were also deposited on the LBSO buffer layers with different thicknesses on the STO substrate, resulting in a phase-pure perovskite PMN–PT layer when grown on a nearly relaxed LBSO template layer. The relaxed LBSO template layer provides a tensile epitaxial mismatch with the PMN–PT. The resulting PMN–PT films show excellent crystalline quality: the fwhm of the ω scan of the (002) reflection is close to that of the used STO substrate and even smaller than found for PMN–PT single crystals.

This work demonstrates a new strategy to grow phase-pure perovskite PMN–PT thin films on STO substrates with a standard miscut, using LBSO as the growth template layer (and as the bottom electrode) with a tensile lattice mismatch with the PMN–PT. Furthermore, it shows the importance of kinetics on the stabilization of the perovskite phase of PMN–PT layers, which is often overlooked in the literature. The outcome of this work also opens the possibility of integrating PMN–PT thin films with Si-based MEMS, employing an STO buffer layer.

## Supporting Information Available

XRD, RHEED, and EDX data of PMN–PT layer on LBSO-coated STO and PPMS and AFM data of LBSO (PDF)
