# Supplementary material for: Stabilizing Perovskite Pb(Mg0.33Nb0.67)O3–PbTiO3 Thin Films by Fast Deposition and Tensile Mismatched Growth Template
Source: ACS Appl Mater Interfaces. 2024 Feb 29;16(10):12744–53. doi: 10.1021/acsami.3c16241 (PMC10941063; doi:10.1021/acsami.3c16241)
Supplement: Supplementary file 1 — am3c16241_si_001.pdf [file am3c16241_si_001.pdf]

Supplementary Information:
Stabilizing Perovskite $Pb(Mg_{0.33}Nb_{0.67})O_3$-$PbTiO_3$ Thin Films by Fast Deposition and Tensile Mismatched Growth Template

Ni, Shu[1]; Houwman, Evert[1]; Gauquelin, Nicolas[2]; Chezganov, Dimitry[2]; Van Aert, Sandra[2]; Verbeeck, Johan[2]; Rijnders, Guus[1]; Koster, Gertjan[1*]

1. MESA+ Institute for Nanotechnology, Faculty of Science and Technology, University of Twente, Enschede 7500 AE, Netherlands

2. Electron Microscopy for Materials Research (EMAT), Department of Physics, University of Antwerp, Antwerpen BE-2020, Belgium; NANOlab Center of Excellence, University of Antwerp, Antwerpen BE-2020, Belgium

Email: g.koster@utwente.nl

S1 Reciprocal space map of LBSO/STO heterostructure

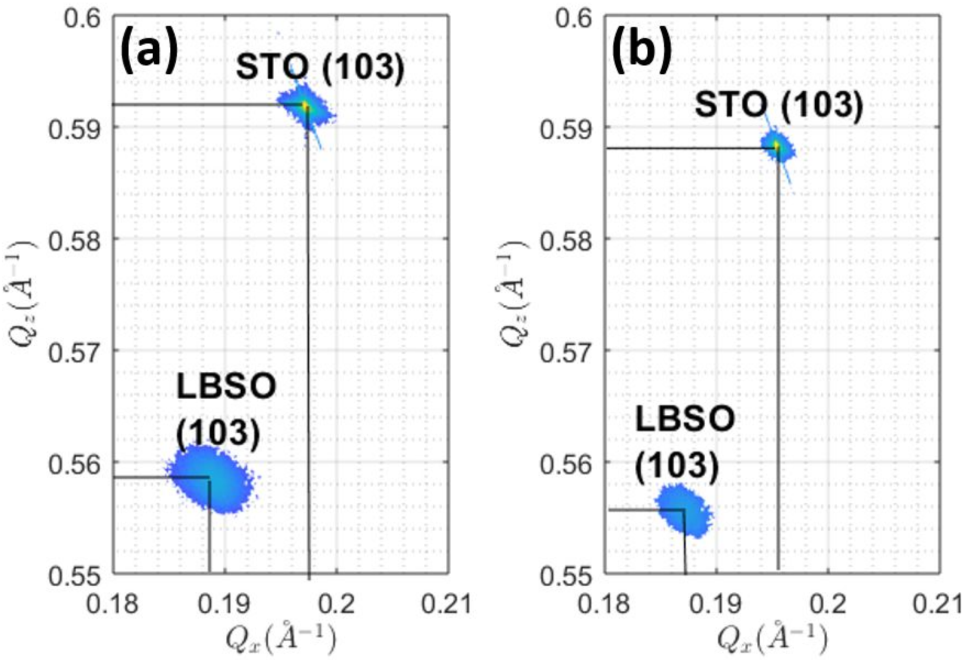

Figure S1 (a) Reciprocal Space Map (RSM) around (103) peaks of LBSO grown on STO at room temperature, (b) idem for LBSO at 600 °C.

Figure S1a and S1b show the Reciprocal Space Maps (RSM) around (103) reflections of 100 unit cell (u.c) thick LBSO on $SrTiO_3$ (STO) at room temperature and the growth temperature of PMN-PT (600 °C), respectively. The deduced room temperature in-plane and

out-of-plane lattice parameters are a=4.079 Å and c=4.139 Å for the LBSO film, and a=c=3.905 Å for the STO substrate. The pseudocubic lattice parameter of LBSO is obtained as a_{pc}=4.099 Å. At the deposition temperature of PMN-PT (600 °C), the deduced in-plane and out-of-plane lattice parameters of LBSO are a=4.112 Å and c =4.160 Å, and therefore a_{pc}=4.127 Å, and a=c=a_{pc}=3.930 Å for the STO substrate. At room temperature, PMN-PT has a pseudocubic lattice parameter around a_{pc}=4.022 Å. Using the thermal expansion coefficient of PMN-PT of 3.2×10^{-6} °C[1,2], the pseudocubic lattice parameter of PMN-PT at the growth temperature is expected to be a_{pc}=4.029 Å, which has a compressive lattice mismatch of -2.5% with respect to the STO substrate and a tensile mismatch of 2.4% with respect to the LBSO film.

S2 EDX chemical composition maps of PMN-PT layers

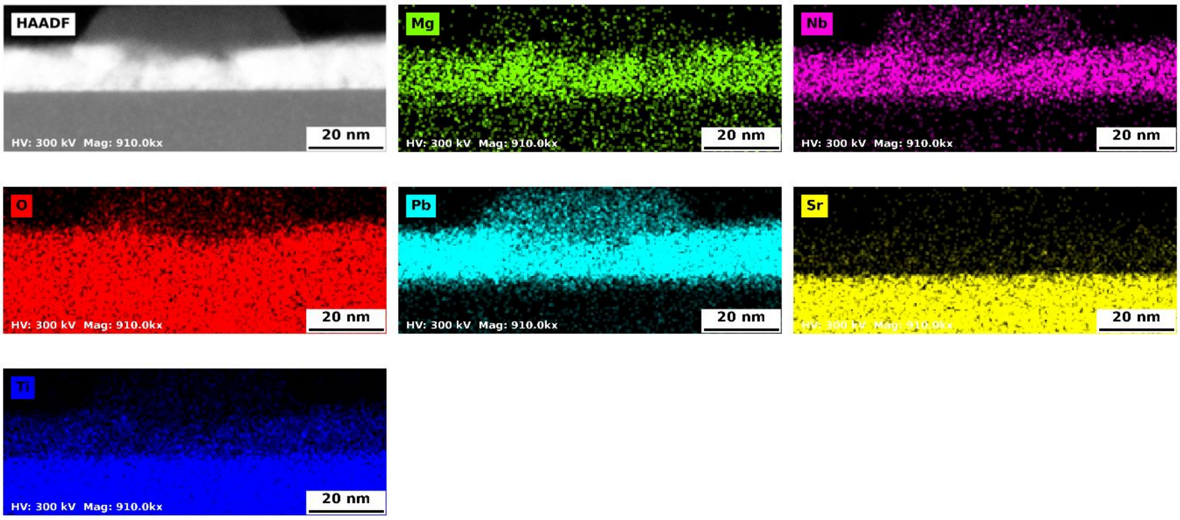

Figure S2 EDX chemical composition maps of 15 nm thick PMN-PT grown at 5 Hz on STO substrate.

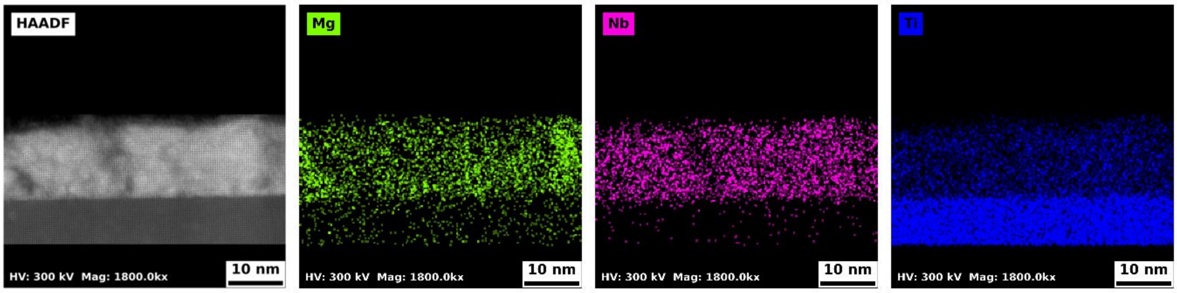

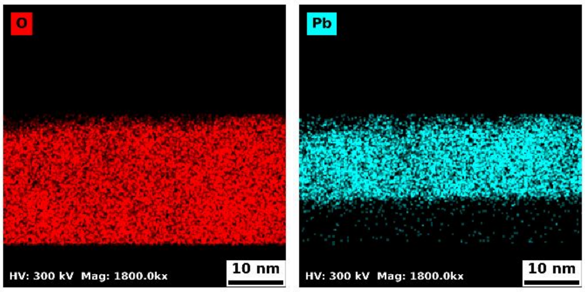

Figure S3 EDX chemical composition maps of 15 nm thick PMN-PT grown at 20 Hz on STO substrate.

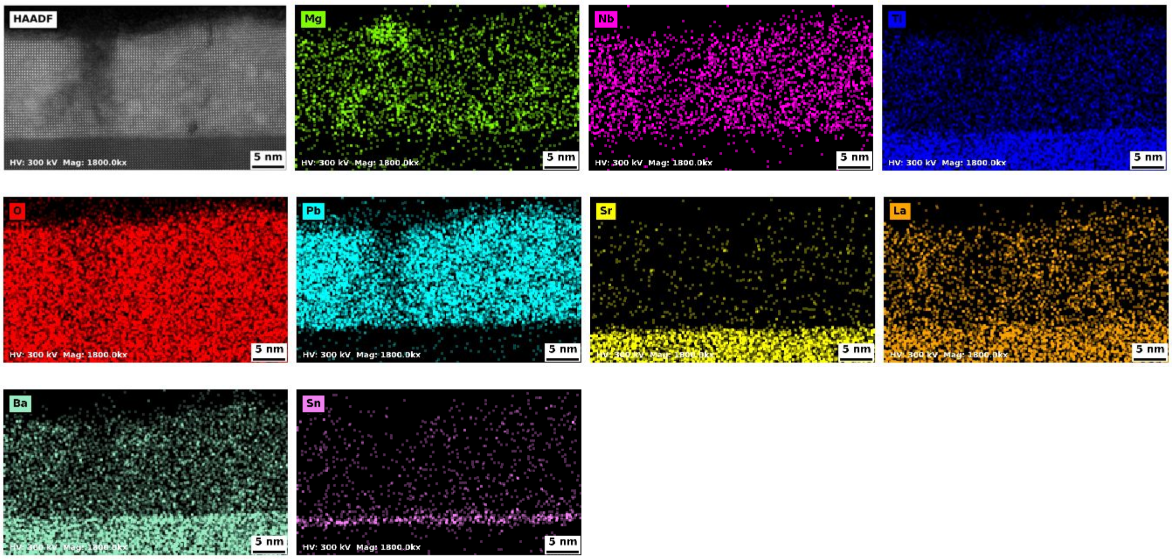

Figure S4 EDX chemical composition maps of 15 nm thick PMN-PT grown on 2 u.c LBSO.

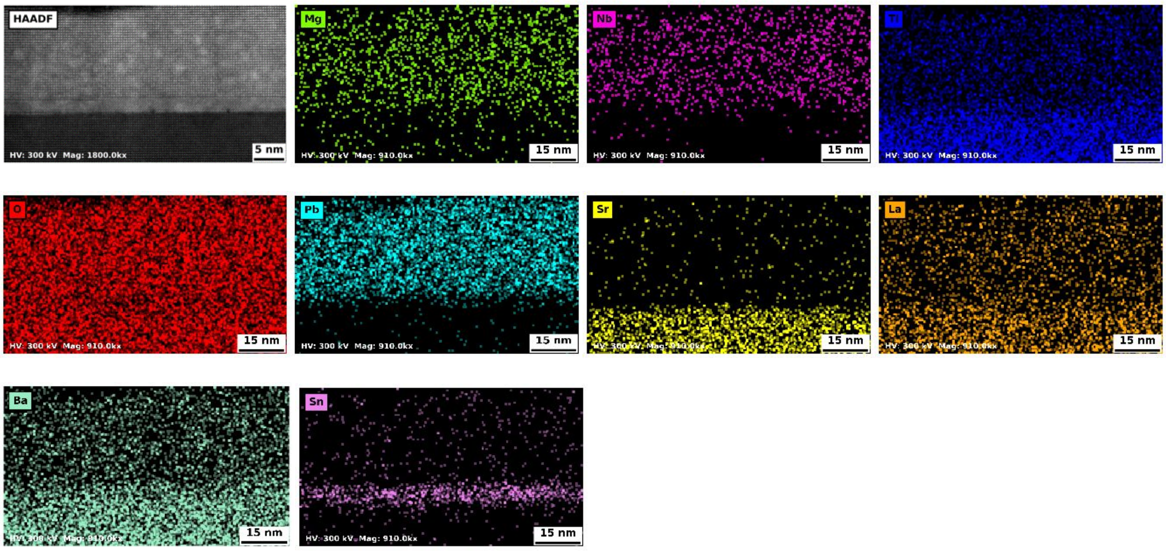

Figure S5 EDX chemical composition maps of 15 nm thick PMN-PT grown on 9 u.c LBSO.

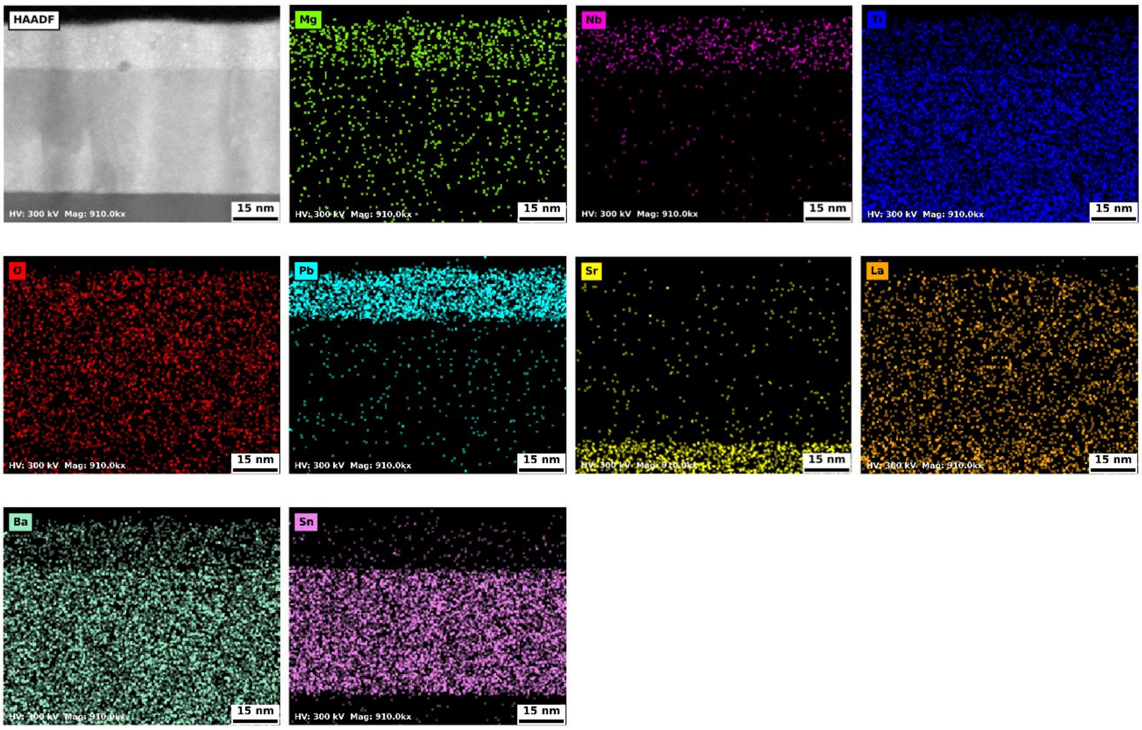

Figure S6 EDX chemical composition maps of 15 nm thick PMN-PT grown on 100 u.c LBSO.

S3 PMN-PT on SRO coated STO

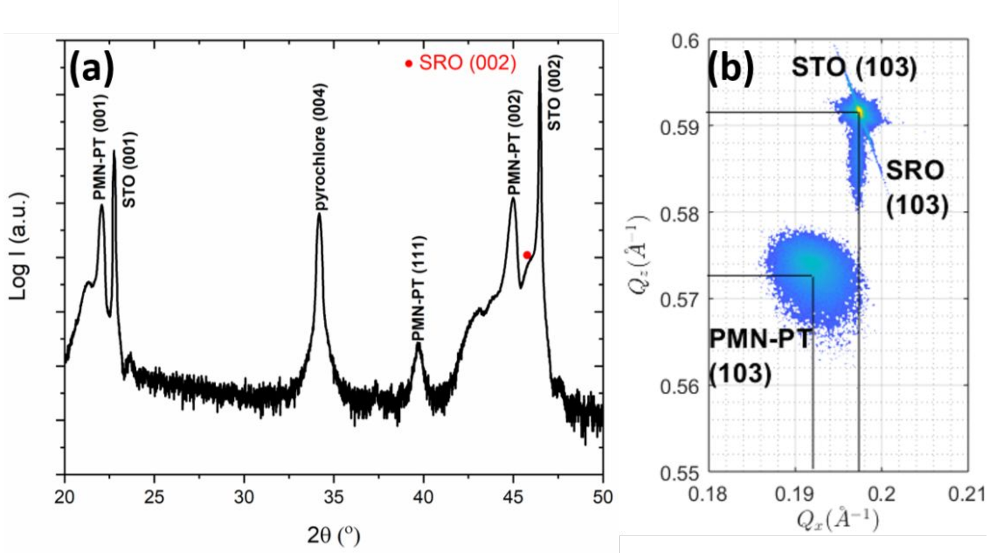

Figure S7 XRD scans of 500 nm thick PMN-PT grown on SRO (20 nm) coated STO grown at 20 Hz (a) $\theta - 2\theta$ scans of 500 nm thick PMN-PT films grown on SRO (20 nm) coated STO (b) Reciprocal space mapping around (103) peaks of 500 nm thick PMN-PT grown on SRO (20 nm) coated STO.

500 nm thick PMN-PT was attempted to be deposited on 20 nm thick $SrRuO_3$ (SRO)

coated STO substrate at 20 Hz. The growth parameters are kept identical with the growth of PMN-PT on STO. The room temperature XRD scan of the resulting film is shown in Figure S7 a indicating that the PMN-PT contains a pyrochlore phase and (111) oriented perovskite PMN-PT. Furthermore, a sharp peak on top of a broad peak is observed for the (002) reflection of PMN-PT, which is similar to the (002) peak profile of 500 nm thick PMN-PT directly grown on STO as shown in Figure 4b in the main manuscript. Figure S4b shows the RSM of the (103) reflections of PMN-PT grown on the SRO coated STO. It is seen that the SRO is fully strained to the STO substrate. The deduced in-plane and out-of-plane lattice parameters of the PMN-PT are a=4.012 Å and c=4.029 Å, respectively. The tetragonality of the resulting PMN-PT is around 1.004 and the pseudocubic lattice parameter is around a_{pc} =4.017 Å. This shows that the PMN-PT is under slight compressive strain and the unit cell volume is somewhat reduced compared to that of bulk single crystal PMN-PT

S4 Surface morphology of LBSO of different thicknesses grown on STO

The 2 u.c LBSO grown on STO (Figure S4a) shows visible step-terrace structure, indicating coherent growth and smooth surface. With increasing thickness, the surface roughness increases and shows more island growth feature. The imprint of the substrate step-terrace structure is absent for 100 u.c (41 nm) thick LBSO. The peak-to-peak height differences are around 0.3 nm for 2 u.c thick LBSO; 0.5 nm for 4, 9, and 15 u.c thick LBSO and 1 nm for 100 u.c. (41 nm) thick LBSO, as given in the inset of the figures.

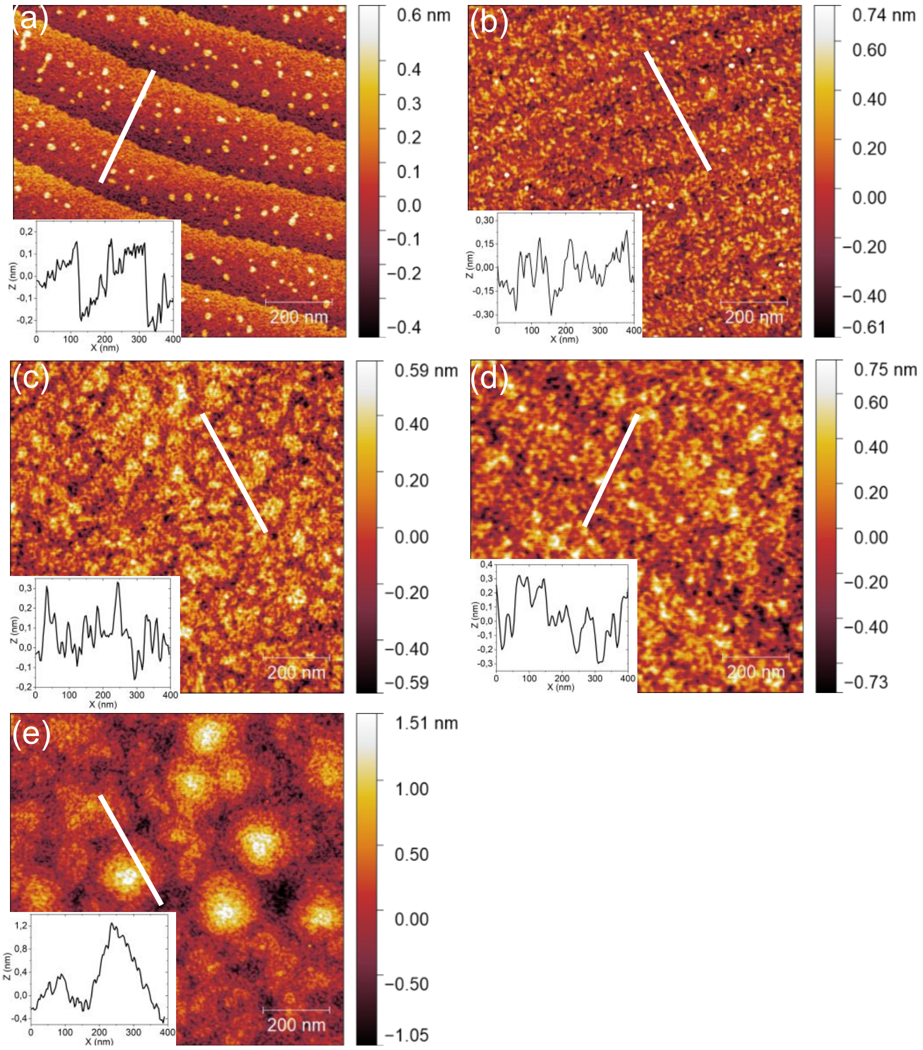

Figure S8 AFM surface morphology and the corresponding cross-section profiles of (a) 2 u.c (b) 4 u.c (c)9 u.c (d) 15 u.c and (e) 100 u.c thick LBSO grown on STO.

S5 Electrical resistivity of LBSO on STO

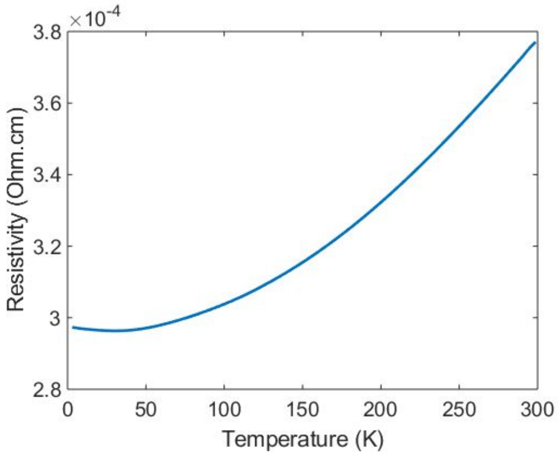

Figure S9 Temperature dependent electrical resistivity of 100 u.c thick LBSO grown on STO.

The electrical properties of 100 u.c. thick LBSO films were measured using Van de Pauw geometry in a Quantum Design Physical Properties Measurement System (PPMS) (Quantum Design). Titanium and gold were deposited at the four corners of the sample to obtain good electrical contacts. The resistivity is measured from 2K to 300K, and the results are shown in Figure S5. It is seen that the resulting LBSO film shows metallic behavior: the resistivity reduces with decreasing temperature. At room temperature, the resistivity of the LBSO films is around $3.78\times10^{-4}\Omega$ cm. Hall measurements were performed at 300 K and the carrier density and mobility of the LBSO layer was measured to be around $4.0\text{x}10^{20}$ cm^{-3} and 41.3 cm^2 V^{-1} s^{-1}.

S6 PMN-PT layers on LBSO coated STO

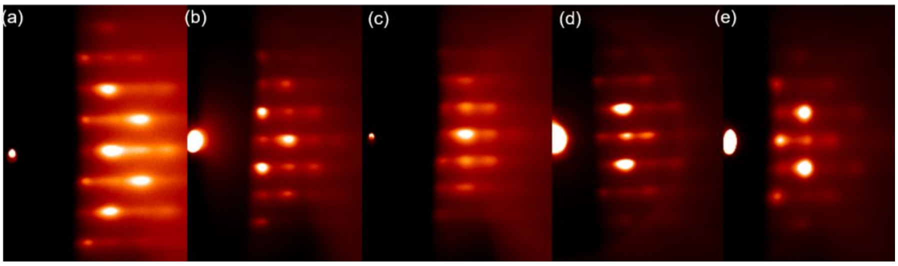

Figure S10 RHEED profiles of 15 nm thick PMN-PT films deposited on (a) 2u.c (b) 4 u.c (c) 9 u.c (d) 15 u.c and (e) 100 u.c thick LBSO layers.

Figure S6 (a-e) shows RHEED patterns of 15 nm thick PMN-PT grown on LBSO layers of 2, 4, 9, 15 and 100 u.c, respectively. It is seen that all PMN-PT films have a pure perovskite phase, which is consistent with the XRD scans shown in Figure 7 in the main manuscript.

To investigate the crystalline quality of the thin PMN-PT films, ω scans were performed on the (002) reflection of PMN-PT grown on LBSO with different thicknesses. Figure S7 and S8 are the results of the scans on 15 and 750 nm thick PMN-PT layers grown on LBSO. The FWHM of the (002) rocking profiles are summarized in Table S1 and S2. It is seen that the FWHM of the (002) reflections are around 0.1° for all 15 nm thick PMN-PT films. However, intensity of the (002) PMN-PT peak first increases with a thicker LBSO and reaches its highest value at 15 u.c, and then reduces slightly for the PMN-PT film grown on LBSO of 100 u.c. The change of the intensity requires further investigation. For the 750 nm thick PMN-PT, the FWHM of the (002) rocking profile decreases significantly from 0.28 to 0.04 when the thickness of the LBSO increases from 2 to 4 u.c. The FWHM of the PMN-PT films deposited on LBSO of 9, 15, and 100 u.c are around 0.04, indicating the superior crystalline quality of the films. The peak intensity of the 750 nm thick PMN-PT films with

respect to the LBSO thicknesses exhibits similar dependence as the 15 nm thick PMN-PT layers.

Table S1 FWHM of (002) reflection of 15 nm thick PMN-PT grown on LBSO layers of different thicknesses

LBSO thickness (unit cell)	FWHM of PMN-PT (002) reflection (°)
2	0.17
4	0.12
9	0.13
15	0.09
100	0.14

Table S2 FWHM of (002) reflection of 750 nm thick PMN-PT grown on LBSO layers of different thicknesses

LBSO thickness (unit cell)	FWHM of PMN-PT (002) reflection (°)
2	0.28
4	0.04
9	0.04
15	0.03
100	0.04

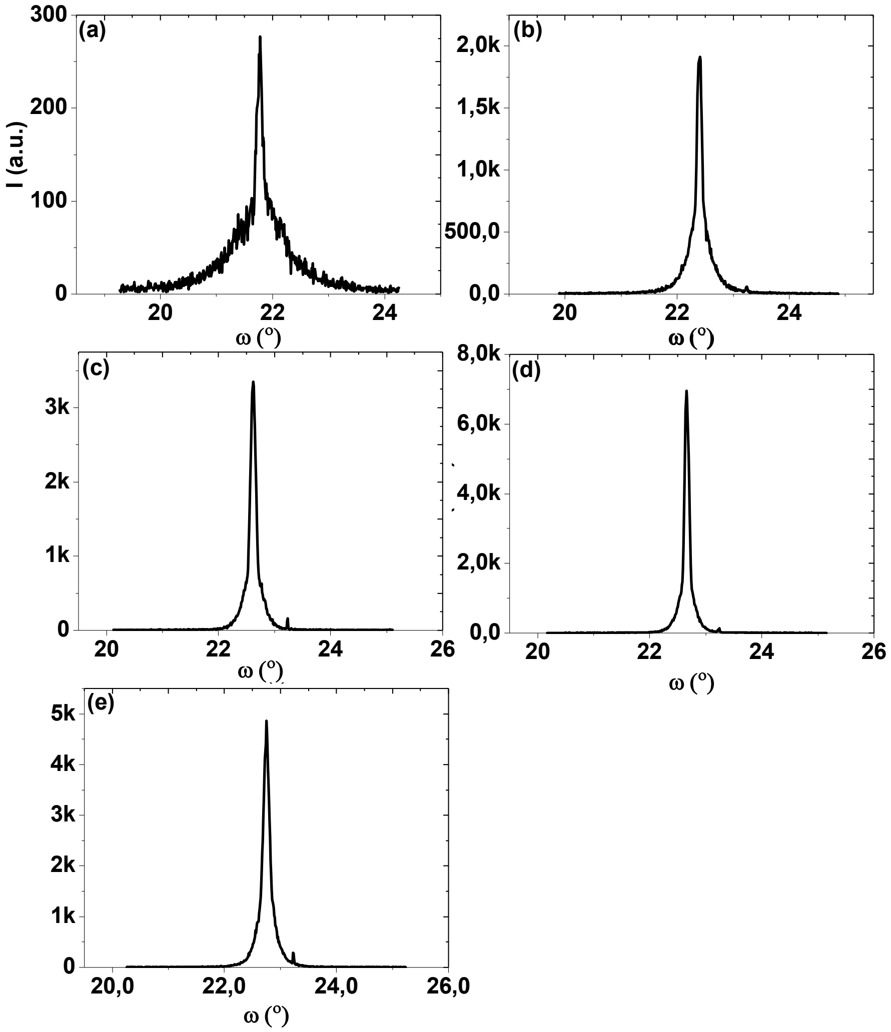

Figure S11 ω scans around (002) reflection of 15 nm thick PMN-PT grown on LBSO buffer layers of (a) 2 u.c (b) 4 u.c (c) 9 u.c (d) 15 u.c (e) 100 u.c

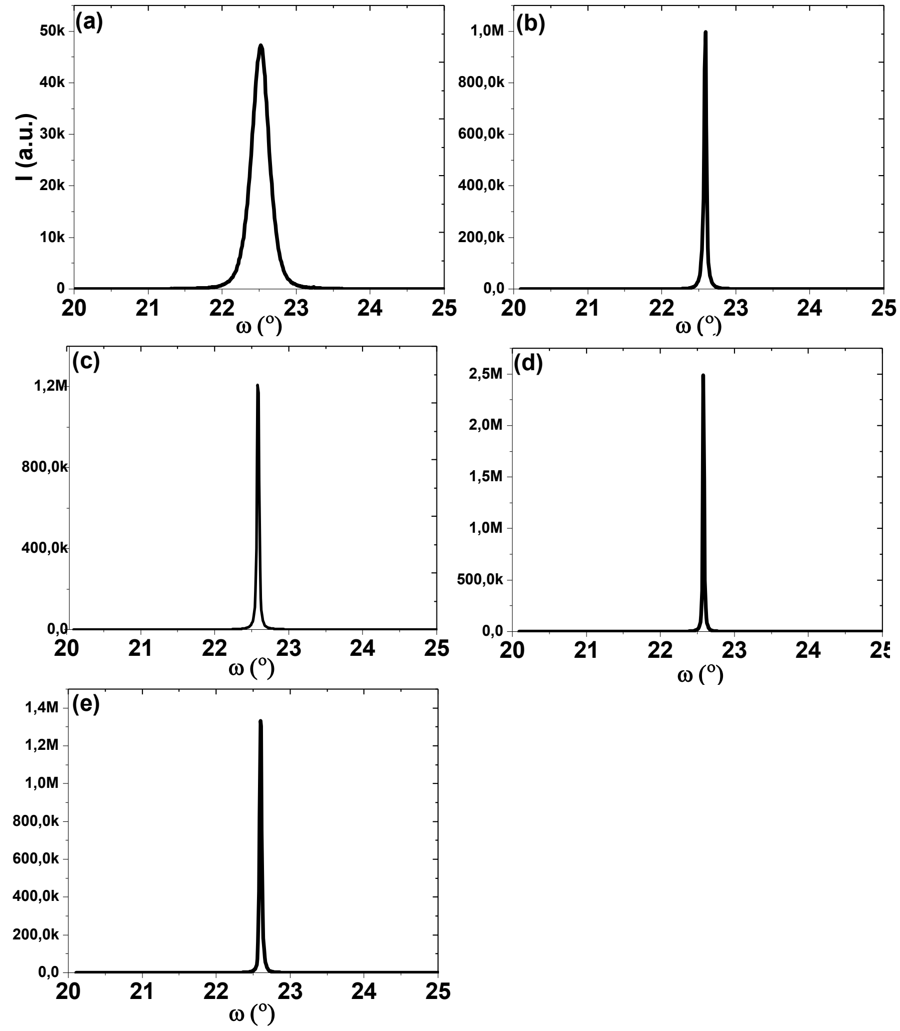

Figure S12 ω scans around (002) reflection of 750 nm thick PMN-PT grown on LBSO buffer layers of (a) 2u.c (b) 4 u.c (c) 9 u.c (d) 15 u.c (e) 100 u.c.

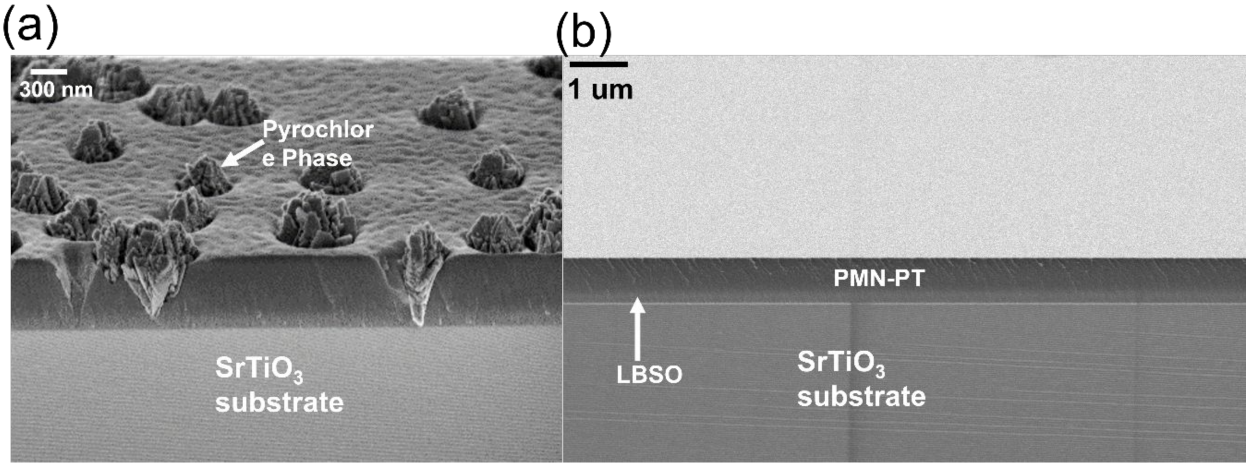

Figure S13 Scanning Electron Microscopy images of PMN-PT layers (a) 500 nm PMN-PT thin film deposited directly on $SrTiO_3$ substrate at 20 Hz. The columnar structures are pyrochlore phases (b) 750 nm phase-pure perovskite PMN-PT layer deposited on 100 u.c LBSO at 20 Hz.

Reference

[1] Wongmaneerung, R., Guo, R., Bhalla, A., Yimnirun, R., & Ananta, S. (2008). Thermal Expansion Properties of PMN–PT Ceramics. *Journal of Alloys and Compounds*, *461*(1-2), 565-569.

[2] Li, Z., Xu, Z., Xi, Z., Xiang, F., & Yao, X. (2007). Thermal Expansion Characteristics in [001]-Oriented PMN-0.32 PT Single Crystals. *Ferroelectrics*, *355*(1), 245-251.
